# Guest Editorial

**Published:** 2016-09-27

**Authors:** Nikhil Srivastava

**Affiliations:** MDS, FICD, FDS-RCPS (Glasg) Professor and Head, Pedodontics and Preventive Dentistry Principal, Subharti Dental College and Hospital Dean, Faculty of Dental Sciences Swami Vivekanand Subharti University Meerut, Uttar Pradesh, India

**Suicidal behavior in children; Can we do something?**

“Suicide is an act that is contrary to what is perhaps the strongest of human instincts survival.” *- The Lancet, 2012*

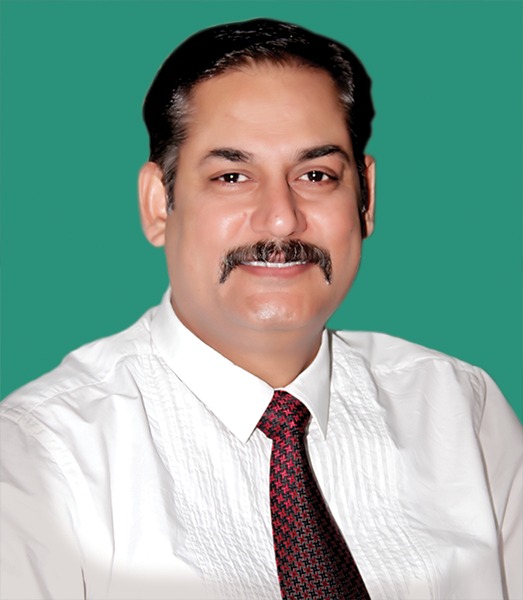



Suicide is essentially defined as death caused by self-directed injurious behavior with any intent to die as a result of this behavior (American Psychological Association). An equally serious issue is suicidal attempt which means an intention to die as a result of suicidal behavior. Shockingly, it is the 3rd leading cause of death for young people between the ages of 10 to 24 years. Globally, boys have a greater chance of committing suicide than girls. According to ‘The Lancet’ (2012), suicide death rates in India are among the highest in the world!

In order to discuss this growing and very serious problem, it is important to understand why suicides occur. The common causes for suicides in children and adolescents range from child abuse, bullying in school, a sense of hopelessness and helplessness, alcohol/drug abuse, mental disorders, and access to firearms or dangerous substances. The high suicide rates may come from “the greater likelihood of disappointments when aspirations that define success and happiness are distorted or unmet by the reality faced by young people in a rapidly changing society” according to Dr Vikram Patel in an editorial printed in The Hindu. Conversely, strong connections to family, friends, and community as well as cultural and spiritual beliefs act as protective factors for suicide. Just like earthquakes and tsunamis, a key factor in suicide prevention is identifying early warning signs in children and adolescents. Specific warning signs include talking about dying, a change in personality, behavior or sleep patterns which may be seen as difficulty in concentration, sadness, insomnia or anxiousness. The greatest predictor of a future suicide is a previous suicidal attempt and such children need to be carefully monitored.

The question facing us is - what can we do about it? Suicide prevention involves a multidisciplinary approach involving parents, teachers and other relevant adults in a child’s life as well as health professionals and the legal system (Radhakrishnan and Andrade, 2012). As health professionals, our primary role is to increase awareness among parents and school teachers as suicide is a topic that is surrounded by social taboo and people are reluctant to acknowledge that suicide is a real problem our children are facing today. Suicide prevention helplines are also available in India now such as the ones by AASRA or OneLife foundations which offer a 24x7 helpline number (AASRA:91-22-27546669 and OneLife:91-78930 78930). This information can be passed onto schools and to parents as well as child patients. September 10 is the World Suicide Prevention Day and acknowledging this day in hospitals and schools may help to highlight the importance of suicide prevention. Good communication channels and a holistic approach to child well being may go a long way in preventing suicide in children and adolescents and avoiding devastating consequences for the entire family and community.

**Nikhil Srivastava**

MDS, FICD, FDS-RCPS (Glasg)

Professor and Head, Pedodontics and Preventive Dentistry

Principal, Subharti Dental College and Hospital

Dean, Faculty of Dental Sciences

Swami Vivekanand Subharti University

Meerut, Uttar Pradesh, India

